# Biophysical Description of Multiple Events Contributing Blood Leukocyte Arrest on Endothelium

**DOI:** 10.3389/fimmu.2013.00108

**Published:** 2013-05-15

**Authors:** Philippe Robert, Dominique Touchard, Pierre Bongrand, Anne Pierres

**Affiliations:** ^1^Laboratoire Adhésion and Inflammation, Aix-Marseille UniversitéMarseille, France; ^2^Institut National de la Santé et de la Recherche MédicaleMarseille, France; ^3^Centre National de la Recherche ScientifiqueMarseille, France; ^4^Laboratoire d’Immunologie, Hôpitaux de Marseille, Hôpital de la ConceptionMarseille, France

**Keywords:** adhesion, ligand-receptor interaction, bond strength, integrin, clustering, avidity, dynamics, LAD-III

## Abstract

Blood leukocytes have a remarkable capacity to bind to and stop on specific blood vessel areas. Many studies have disclosed a key role of integrin structural changes following the interaction of rolling leukocytes with surface-bound chemoattractants. However, the functional significance of structural data and mechanisms of cell arrest are incompletely understood. Recent experiments revealed the unexpected complexity of several key steps of cell-surface interaction: (i) ligand-receptor binding requires a minimum amount of time to proceed and this is influenced by forces. (ii) Also, molecular interactions at interfaces are not fully accounted for by the interaction properties of soluble molecules. (iii) Cell arrest depends on nanoscale topography and mechanical properties of the cell membrane, and these properties are highly dynamic. Here, we summarize these results and we discuss their relevance to recent functional studies of integrin-receptor association in cells from a patient with type III leukocyte adhesion deficiency. It is concluded that an accurate understanding of all physical events listed in this review is needed to unravel the precise role of the multiple molecules and biochemical pathway involved in arrest triggering.

## Introduction

Immune cells such as lymphocytes or phagocytes can bind to specific blood vessel areas and further migrate toward peripheral tissues. This allows memory lymphocyte patrolling throughout the organism to detect invading foreign material. Also, this allows endothelial cells of inflamed areas to trigger the arrest of blood leukocytes that are flowing in a resting state. Basic mechanisms have been elucidated during the early nineties (Lawrence and Springer, 1991; von Andrian et al., 1991; Springer, 1994), leading to a general paradigm that remains valid (Ley et al., 2007): leukocytes move with a velocity of several millimeters/second imposed by the blood flow (Atherton and Born, 1972). The earliest event is cell-surface tethering by specialized membrane receptors (Lawrence and Springer, 1994) such as P-selectin (CD62-P) on stimulated endothelial cells or L-selectin that is concentrated on the tip of leukocyte microvilli. Cells then display a somewhat jerky displacement (5–10 μm s^−1^) called rolling. This is due to the rapid formation and dissociation of bonds such as are formed between endothelial E- and P-selectins and lymphocyte-associated ligands comprising P-selectin glycoprotein ligand 1 (PSGL-1), E-selectin ligand 1 (ESL-1), and the hyaluronan receptor CD44 (Hidalgo et al., 2007). Tethering and rolling may also be driven by the interaction between vascular cell adhesion molecule 1 (VCAM-1) expressed on properly stimulated endothelial cells and α4β1 (VLA-4, CD29dCD49) expressed on some leukocyte populations (Alon et al., 1995). A key property of bonds mediating rolling is their capacity to resist hydrodynamic forces of several tens of piconewtons for several tenths of a second (Evans et al., 2001, 2004). Rolling does not require any active cell participation since it may be reproduced with fixed cells (Lawrence and Springer, 1993) or with cell-free systems (Brunk et al., 1996). A likely explanation of rolling jerkiness is that at a given time a leukocyte is bound by a few or even a single bond and each bond rupture event results in a discrete forward displacement. Indeed, rolling velocity is strongly correlated to the bond dissociation rate (Alon et al., 1997).

The initial step of rapid rolling may be followed by an intermediate phase of “slow rolling” with more than twofold velocity decrease. This may result from a partial activation of lymphocyte function associated 1 integrin (LFA-1, CD11aCD18) enabling it to interact with intercellular cell adhesion molecule 1 (ICAM-1, CD54) expressed by endothelial cells (Jung et al., 1998). LFA-1 activation may be induced by E-selectin interaction with PSGL-1 (Kuwano et al., 2010) or CD44 (Yago et al., 2010).

Other phenomena were found to contribute the following arrest phase: the pulling force applied on cell-surface receptors may generate membrane tubes of up to 40 μm length (Schmidtke and Diamond, 2000), thus decreasing the force applied on bonds as explained below. Also, it was recently shown that the tethers formed on neutrophils could wrap around rolling cells and display a “stepwise peeling” through patches of PSGL-1 molecules interacting with substrate P-selectin (Sundd et al., 2012). The authors suggested that this particular behavior might be responsible for the neutrophil capacity to roll at extremely high shear rates.

Arrest is mainly triggered by the complete activation of leukocyte integrins such as LFA-1 or VLA-4, enabling them to firmly bind endothelial ligands such as ICAM-1 or VCAM-1 respectively, as reviewed in this research topic (Chigaev and Sklar, 2012; Lefort and Ley, 2012). Subsecond integrin activation (Grabovksy et al., 2000; Alon and Dustin, 2007) is triggered by endothelium-bound chemoattractants that often belong to the chemokine family (Zlotnik and Yoshie, 2012). Thus, the same receptor family may be involved in directing cell locomotion and triggering arrest under shear flow (Campbell et al., 1998). The specificity of leukocyte species and arrest location is imparted by a particular combination of chemokines, adhesion molecules, and stimulation pathway (Rot and von Andrian, 2004). Following arrest, leukocytes may start crawling toward endothelial junctions and transmigrate toward surrounding tissues (Schenkel et al., 2004).

A current challenge is to understand the role of all involved molecules and signaling pathways. Here we shall describe the elementary physical events contributing the transition from rolling motion to LFA-1-mediated firm arrest. Indeed, a detailed understanding of physical constraints should help us understand the rationale of all cell processes contributing arrest. General concepts will be illustrated by addressing a specific problem: relating kindlin-3 deficiency to functional defects in LAD-III patients.

A prerequisite for assessing the use and significance of elementary events such as integrin clustering or membrane topographical reorganization is to build a quantitative scheme of the arrest phenomenon as a physical process.

## Physical Background

To estimate the intensity and effect of forces applied on leukocytes under flow, we need a simple model of cells as physical objects.

### Mechanical and geometrical properties of blood leukocytes

Micrometer-scale leukocyte rheological properties were studied by monitoring the deformation of cells sucked into micropipettes with controlled pressure (Evans and Yeung, 1989). Neutrophils behaved as viscous liquid spherical droplets (about 10^−5^ Pa.s viscosity and 8 μm diameter) surrounded by a membrane under tension (∼3.5 × 10^−5^ N m^−1^). This is a minimal model (Herant et al., 2003). First, cells are composite objects. Thus, nuclear and cytoplasmic properties may be widely different. Secondly, applying mechanical forces may initiate active mechanical responses (Horoyan et al., 1990). However, this model may be relevant to the initial phase of leukocyte arrest under flow.

The structural basis of cell mechanical properties was studied with electron microscopy. Leukocytes are surrounded by a fairly inextensible lipid bilayer with numerous folds appearing as finger-like structures called microvilli or ridge-like folds (Bruehl et al., 1996; Shao et al., 1998). The average length is ∼0.3 μm and diameter or thickness is ∼0.2 μm. When pulling at microbeads bound to neutrophil microvilli, Shao et al. (1998) found that forces lower than 34 pN triggered elongation with a proportionality Hook parameter of 43 pN μm^−1^, while forces higher than 61 pN separated the plasma membrane from underlying cytoskeleton, thus generating tethers with an elongation rate proportional to the applied force. More recently, based on the brownian motion of microspheres bound to the tips of microvilli, Yao and Shao (2007) estimated the flexural stiffness at 7 pN μm^−1^.

Thus, membrane unfolding is required for a spherical cell to spread on a surface. The maximum increase of apparent cell area after complete unfolding is ∼50–100% (Evans and Yeung, 1989; Bruehl et al., 1996). Further area increase may require fusion of intracellular vesicles with plasma membranes, which may occur a few minutes after the onset of spreading (Gauthier et al., 2009).

We shall use this information to estimate the constraints experienced by a blood leukocyte made to stop in a specific area in blood vessels.

### Effect of flow on blood leukocytes

**Figure 1:**
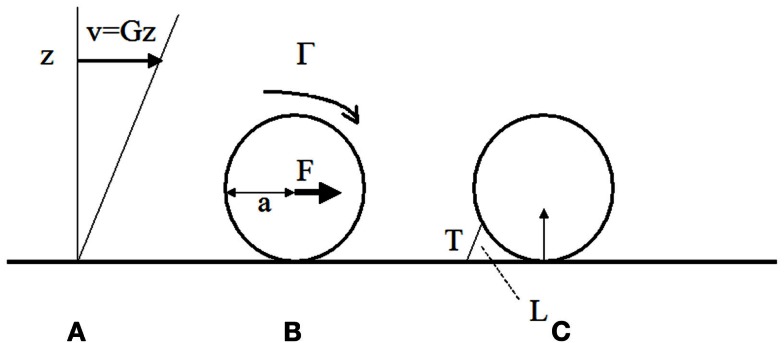
**Hydrodynamic forces on cells bound to blood vessel walls**. **(A)** In a laminar viscous shear flow near a plane, the blood velocity at any point near the wall is parallel to the plane and equal to the distance z to the wall times the wall shear rate G (in s-1). The shear stress is the shear rate times the fluid viscosity μ (μ ∼ 0.001 Pa.s in aqueous medium). It represents the viscous force applied by the fluid on an unit area on the wall. **(B)** The fluid exerts on a sphere of radius bound to the wall a total force *F* ∼32 μa^2^G and a torque G ∼ 11.9 μa^3^G (Goldman et al., 1967). **(C)** if the sphere is maintained at rest by a single bond of length L and the contact between the surface and the wall is assumed to be frictionless, the tension T on the bond is ∼ 31 μa^2^G (a/L)^1/2^ (Pierres et al., 1995).

Blood flow is very different in millimeter diameter arteries and micrometer-diameter capillary vessels. Here, we shall focus on postcapillary venules with a diameter of several tens of micrometers, since they are a typical region of leukocyte arrest. As recalled on Figure 1A, the blood velocity near the vessel wall at any point M is parallel to the vessel axis and close to *G*.*z*, where *z* is the distance between M and the wall, and *G* (in second^−1^) is called the wall shear rate. Typical wall shear rates of a few hundreds of s^−1^ are found in postcapillary venules (Atherton and Born, 1973). The contact time between microvillus receptors and endothelium is thus lower than 1 ms (Zhao et al., 2001). This is the time allowed for initial tethering of cells to the endothelial surface. What happens then?

The force applied on a 8 μm diameter leukocyte when the shear rate is 200 s^−1^ is ∼102 pN (Figure 1B). The force on a P-selectin-PSGL-1 couple of 80 nm length may be sevenfold higher than the force on the cell (Figure 1C; Pierres et al., 1995). If the bond is located at the tip of a protrusion of 0.3 μm length, the force will be 3.7-fold higher than the force on the cell. This may induce tether formation if the receptor is not firmly anchored to the cell cytoskeleton. This was actually observed (Schmidtke and Diamond, 2000; Sundd et al., 2010, 2012). Thus a few bonds located at the tip of microvilli may not be sufficient to immobilize a leukocyte. Repeated bond formation and rupture will generate a rolling motion. Molecular contacts between leukocyte receptors and endothelial ligands may then last several tens of milliseconds rather than milliseconds for freely flowing cells. This may permit integrin-mediated attachments.

Thus, stopping a leukocyte on the blood vessels will need to resist local pulling forces between 100 and 700 pN. We need know how many adhesion receptors are needed to fulfill this task. Results accumulated during the last two decades may provide a clear answer to this question.

### New methods and concepts provide us with quantitative information on the properties of bond formation and dissociation between surface-attached molecules

#### Inability of the conventional framework to account for interactions between surface-attached molecules

As previously reviewed (Bongrand, 1999; Zhu et al., 2002; Robert et al., 2007), the interaction between two molecules A and B in solution is well accounted for by two numbers, the association rate *k*_on_ and dissociation rate *k*_off_:

(1) $$\text{A + B}\underset{k_{\text{off}}}{\overset{k_{\text{on}}}{⇄}}\text{AB}$$

*d*[AB]/*dt* = *k*_on_ [A][B] − *k*_off_ [AB], the ratio *k*_on_/*k*_off_ is the affinity constant *K*_a_.

However, this conventional framework could not account for interactions between membrane-bound receptors and ligands: Firstly, bonds formed between surface-bound molecules are often subjected to external forces, and until recently no information was available on the effect of forces on bond lifetime. Secondly, as emphasized earlier, even the dimension of association rate between surface-bound molecules (corresponding to so-called 2D conditions) is different from the dimension of conventional (3D) association rates as defined in Eq. 1 (Pierres et al., 2001). Thirdly, 2D conditions impose special constraints on multivalent associations. We shall address these points sequentially.

#### Rupture of bonds between surface-attached molecules

**Figure 2:**
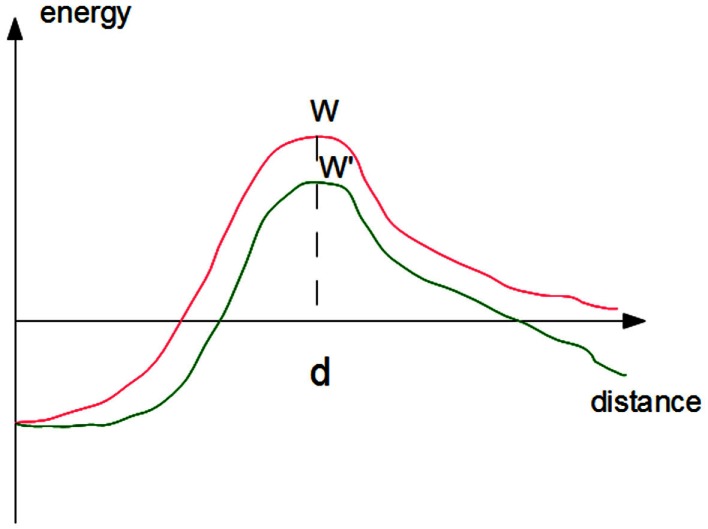
**Effect of forces on the kinetics of bond rupture**. The simplest approximation consists of representing the free energy of a ligand-receptor complex as a simple function of the distance between ligand and receptor surfaces (red curve). Rupture requires the crossing of an energy barrier of height *W*. The rupture rate may be viewed as the product of the frequency of attempts at crossing times the success probability that is proportional to Boltzmann’s factor exp(−*W*/*k*_B_T). Applying a force will decrease the barrier height by the product *F*.*d*, i.e., the force times the distance between the barrier and the equilibrium distance, thus multiplying the escape frequency by exp(*Fd*/*k*_B_T).

During the last two decades, experiments based on laminar flow chambers (Kaplanski et al., 1993; Pierres et al., 1995), atomic force microscopes (Florin et al., 1994), the biomembrane force probe (Merkel et al., 1999), or optical tweezers (Nishizaka et al., 1995) allowed us to study single-bond formation and rupture between surface-attached molecules subjected to controlled forces. Bond rupture under force often followed a simple formula (Chen and Springer, 2001; Evans et al., 2010) previously suggested by Bell (1978):

(2) $$k_{\text{off}}\left(F\right)=k_{\text{off}}\left(0\right)\;\exp\left(F\text{/}F^{0}\right)$$

where *k*_off_(*F*) is the rupture frequency (in s^−1^) of a single-bond subjected to a distractive force F. A simple interpretation of this formula can be obtained by viewing bond rupture as the passage of a molecular complex AB from a bound state at zero separation to a free state that is reached by crossing an energy barrier of height *W* at separation distance *d* (Figure 2). According to Boltzmann’s law, the probability of barrier-crossing should be proportional to exp(−*W*/*k*_B_T), where *k*_B_ is Boltzmann’s constant and T is the absolute temperature. Applying a force *F* will decrease *W* by the product *Fd* (Figure 2) thus multiplying the rupture frequency *k*_off_ by exp(*Fd*/*k*_B_T). Bell estimated at 0.5 nm the order of magnitude of parameter d for an antigen-antibody interaction corresponding to the depth of an antibody binding site, leading to an estimate of ∼8 pN for parameter *F*°= *k*_B_T/*d*. More detailed discussion may be found in a number of papers following Eyring’s (1935) and Kramer’s (1940) seminal papers (Hänggi et al., 1990; Evans and Ritchie, 1997; Dudko et al., 2008). The rupture frequency and force coefficient F° for a number of receptors including selectins, integrins, cadherins, or antibodies were often on the order of 1–100 pN and 0.01–10 s^−1^. Depending on molecule conformation, the force-free rupture frequency of LFA-1/ICAM-1 bond varied between 0.008 and 2 s^−1^, with a force coefficient of 7–10 pN (Evans et al., 2010). However, the above results are only an approximation and single molecule studies confirmed that bond rupture is a complex process requiring multiple barrier-crossing events (Pierres et al., 1995; Merkel et al., 1999; Derenyi et al., 2004).

The catch-bond phenomenon, which is highly relevant to leukocyte-endothelium interaction, was predicted on the basis of thermodynamical reasoning by noticing that a disruptive force might decrease bond rupture frequency *k*_off_, although it had to decrease binding affinity *k*_on_/*k*_off_. Bonds displaying such a strange behavior were dubbed “catch bond,” in contrast with “ordinary” bonds that were called “slip bonds,” responding to disruptive forces with increased rupture frequency (Dembo et al., 1988). A few years later, it was reported that L-selectin-mediated rolling required a minimal shear level, suggesting that L-selectin might form catch bonds (Finger et al., 1996). More recently, it was demonstrated with flow chambers that a lectin-like bacterial adhesin formed catch bonds (Thomas et al., 2002), and a similar property was demonstrated on P-selectin/PSGL-1 interaction with both flow chamber and atomic force microscopy (Marshall et al., 2003): Bond lifetime displayed a fairly sharp maximum in presence of a pulling force close to 30 pN. P-selectin/PSGL-1 thus displayed catch-bond behavior in presence of a force ranging between 0 and 30 pN. Theoretical studies led to the conclusion that actual biomolecules interactions are much more complex that sketched on Figure 2. Thus, a catch-bond behavior might be accounted for by the existence of two dissociation pathways (Pereverzev et al., 2005).

#### Formation of bonds between surface-attached molecules

The rate of bond formation between two surfaces bearing known receptors and ligands cannot be derived from a “2-dimensional on-rate constant” since it is dependent on a number of parameters that are extrinsic to the receptor and ligand, including distance between surfaces, lateral mobility of receptors and ligands, length and flexibility of the links between binding sites and membranes, and behavior of surrounding molecules. First, it was suggested that the 3D *k*_on_ (a number expressed in μm^2^ molecule^−1^ s^−1^) had to be replaced with a function *k*_on_(*d*) representing the frequency (in s^−1^) of bond formation between a ligand and a receptor molecules maintained at distance *d* (Pierres et al., 1996). The function *k*_on_(*d*) could in principle be derived experimentally by simultaneous determination of the binding frequency of receptor-bearing microspheres and ligand-coated surfaces and microsphere-to-surface distance (Pierres et al., 1998). However, other experiments show that this seemingly straightforward method may be difficult to use. Indeed, robust receptor-ligand association may not be immediate, and require a non-negligible amount of time for progressive crossing of barriers from less stable to more stable binding states (Pierres et al., 1995; Marshall et al., 2005; Pincet and Husson, 2005). This point was addressed experimentally in a model system (Robert et al., 2009): The formation of an ICAM-anti-ICAM-1 bond required a minimal contact time of about 10 ms to resist a disruptive force of order of 100 pN during at least 200 ms. This challenges the current framework used to describe bond formation (Eq. 1): the probability of bond formation between a ligand and a receptor is not proportional to the contact time. It is 0 if contact is shorter than some threshold, and 1 above this threshold. The threshold is dependent on the sensitivity of bond detection. More experiments are needed to check the relevance of these results to integrin-ligand associations. This is made more difficult to study experimentally by the dependence of integrin conformation on interactions with underlying membranes. However, since antigen-antibody association is very rapid, it is likely that kinetic effects may be found on most biological systems.

#### Difficulty of relating multivalent interactions to monovalent interactions when surface-bound molecules are considered

Theoretical studies (Seifert, 2000) have long revealed the difficulty of relating the properties of multivalent attachments to single bonds. This difficulty is a consequence of two important processes: force-sharing and rebinding. This point can be made easier to grasp by comparing the lifetime of attachments mediated by one or two identical bonds.

First, let us consider the effect of an external force F: if the force is equally shared between both bonds, the lifetime of each bond will be divided by exp(*F*/2*F*°), where *F*° is the aforementioned force constant. After the rupture of a first bond, the whole force will be applied on the remaining one, thus inducing rapid failure. The dissociation rate of the divalent attachment will thus vary as exp(*F*/2*F*°). In absence of force-sharing, the force is expected to divide attachment lifetime by exp(*F*/*F*°). As a numerical example, a disrupting force of 40 pN is expected to reduce the lifetime of a single-bond attachment mediated by an integrin of force constant F°∼ 7 pN by 300, while the lifetime of a force-sharing divalent attachment will be reduced by only 17.

The importance of rebinding may be still more impressive. Let us consider an attachment involving high affinity receptors such that bond formation is much more frequent than bond rupture. If a bond has a high probability to reform after spontaneous rupture, provided a second bond maintains surfaces in close contact, the lifetime of a divalent attachment may be nearly infinite, and in any case much higher than that of a monovalent attachment.

Recently, this point was addressed experimentally by comparing the lifetime of monovalent and divalent attachments formed between ICAM-1-coated surfaces and anti-ICAM-1-coated microspheres (LoSchiavo et al., 2012): the proportion of divalent attachments resisting a force of 30 pN for at least 5 s was 3.7-fold higher than that of monovalent attachments. This was due to a combination of force-sharing, bond maturation and rebinding. Importantly, rebinding requires a tight proximity between receptors.

Remarkably, clustering has been recognized by many authors as a key feature of integrin function (Cambi et al., 2006; Selhuber-Unkel et al., 2008; van Zanten et al., 2009), although other experiments were compatible with the assumption that conformational activation of individual molecules might suffice to initiate adhesion in absence of any significant modulation of clustering (Kim et al., 2004).

The physical background we summarized will help us understand the mechanisms of integrin-mediated leukocyte arrest.

## Cell Actions Required for Integrin-Mediated Arrest

**Figure 3:**
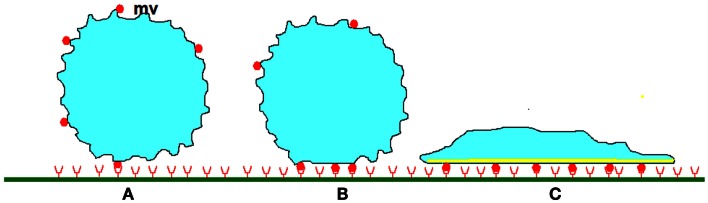
**Stabilization of leukocyte attachment to the blood vessels**. **(A)** When a cell studded with protrusions (mv) of several hundreds of nm length encounters a plane surface, contact between membrane receptors such as selectins or integrins (red disks) and their ligands (red half circles) of total length lower than 50–100 nm can only occur on the tip of protrusions, allowing formation of a low number of bonds. As indicated in text, very high association rates are needed to tether freely flowing leukocytes within a molecular contact shorter than a few milliseconds and initiate rolling. **(B)** Within the following tens of seconds, rolling cells undergo (i) micrometer-scale flattening similarly to liquid droplets encountering a wettable surface, (ii) submicrometer-scale smoothing of microvilli, first due to compressive forces, and possibly later to intracellular signaling triggered by chemokines. (iii) lateral diffusion of membrane receptors that are trapped into the contact area. At some moment, these phenomena induce cell arrest. **(C)** Further attachment strengthening may involve a more extensive increase of contact area as a consequence of spreading, increase of membrane stiffness due to local cytoskeleton reinforcement, and possibly increase of the strength of membrane receptors attachment to underlying cytoskeleton, thus preventing further lateral displacement.

Selectins seem unable to induce durable cell arrests (Lawrence and Springer, 1991) and integrins are required for this purpose. Key events following chemokine-mediated activation (Montresor et al., 2012) are depicted on Figures 3A,B as described below.

### Integrin functional activation

Chemokines were shown to induce *within seconds* an extension of previously bent integrins and opening of binding sites resulting in an affinity increase (Montresor et al., 2012) as a consequence of both increase of binding rate (Vitte et al., 2004; Zhang et al., 2005) and bond lifetime. The binding of immobilized ligands may result in further activation (Alon and Dustin, 2007).

### Membrane alignment

Membrane deformation is required to allow contact between integrin molecules and ICAM-1 ligand. Indeed, the length of the ICAM-1 + LFA-1 couple is about 40 nm, less than the size of the longest microvilli, and LFA-1 is not concentrated on the tip of microvilli in a resting cell (Erlandsen et al., 1993). Molecular contact may thus require at least one of three processes: (i) forces applied on the tip of microvilli may cause significant enlargement and shortening (Sundd et al., 2010). (ii) Chemokines may trigger within seconds ezrin-radixin-moesin dephosphorylation resulting within tens of seconds in microvillus disruption and membrane release (Brown et al., 2003). (iii) Membrane release may enhance transverse membrane undulations as reported at interfaces between glass coverslips and immune cells microscopy (Zidovska and Sackmann, 2006; Pierres et al., 2008; Crétel et al., 2011). Early reports done with electron microscopy (Foa et al., 1988) or fluorescence microscopy (Dustin, 1997) demonstrated submicrometer membrane alignment within minutes (Foa et al., 1988) or even tens of seconds (Dustin, 1997) following cell-surface contact. More recent studies done with interference reflection microscopy showed that the initial attachment of monocytic cells to adhesive surfaces was followed within a minute by progressive interaction tightening that might be interpreted as a nanometer scale alignment of interacting surfaces (Pierres et al., 2002, 2008).

### Lateral redistribution of integrins

#### Integrin alignment with ligands on opposing surfaces

Integrins likely need lateral mobility to align along ligands on opposing surfaces, and the mobility requirement may be higher as lower ligand density (Chan et al., 1991). A positive correlation between lymphocyte adhesiveness to ICAM-1-coated surfaces and LFA-1 membrane mobility was reported (Kucik et al., 1996). More recently, Bakker et al. (2012) concluded that monocytes required a mobile population of surface integrins to adhere to ICAM-1-coated surfaces under static or flow conditions.

The difficulty of relating integrin-cytoskeletal association to cell adhesiveness (Lub et al., 1997) may be due to (i) heterogeneity of mobilities of LFA-1 molecules on a given cell, (ii) contradictory need for mobility (to allow ligand-receptor contact) and integrin-cytoskeleton attachment (to ensure mechanical strength), (iii) dependence of integrin-cytoskeleton interaction on cell differentiation and activation status (Cairo et al., 2006).

#### Integrin clustering

Since the lifetime of a newly formed LFA-1/ICAM-1 bond may be quite short if full activation has not been triggered, a pre-clustering of LFA-1 molecules might strongly enhance the duration of initial attachment and allow for the formation of additional bonds. This may be less necessary if integrins are in a fully activated state. Note that the precise chronology of clustering remains controversial. Studies made on phagocytes strongly suggest that integrin clustering preceded ligand binding (Detmers et al., 1987; Cambi et al., 2006; van Zanten et al., 2009). Other authors concluded that the binding of multivalent ligands was required to induce a clustering of lymphocyte integrins (Kim et al., 2004).

*In conclusion*, integrin-mediated cell arrest likely results from a combination of conformational changes (within seconds), nanotopographical membrane rearrangement to allow contact with ligand-bearing surfaces (within tens of seconds), and lateral diffusion of integrin molecules to align along ligands, form clusters, or both. However, other experiments suggest that initial arrest is followed within minutes by an attachment strengthening phase including several actions, as described below.

### Membrane strengthening and active spreading

Arrest stabilization may include at least three concomitant processes (Figure 3C) That we shall describe separately.

#### Reinforcement of cell stiffness in contact area

Monitoring cell-surface detachment under flow revealed significant cell deformation during detachment (Mège et al., 1986; Cao et al., 1998). Flow induced detachment is a peeling process, with a sequential detachment of membrane stripes involving a few bonds. The rupture force therefore increases in parallel with membrane stiffness (Evans, 1985). This reasoning is supported by experimental evidence (Rees et al., 1977; Badley et al., 1981). This supports the functional importance of microfilament concentration in cell-surface contact areas (André et al., 1991).

#### Reinforcement of integrin anchoring to underlying cytoskeleton

Strong integrin-mediated cell attachment requires that integrins be strongly attached to the cell-surface. Microfilaments indeed enhanced integrin-mediated attachment in some experiments (Lub et al., 1997). Thus, integrin-cytoskeleton interaction may be a multiphasic, time-dependent process: initial integrin release should favor alignment with ligand and clustering, binding of ligand-attached integrins to cytoskeletal elements would then strengthen overall attachment. This is consistent with the multiplicity of integrin states (free versus immobile, isolated versus clustered) on the cell membrane (Cairo et al., 2006).

#### Increase of cell-surface interaction area through active spreading

Spreading may follow and markedly stabilize cell adhesion when this is mediated by suitable receptors (Pierres et al., 2002). A frequent consequence of integrin-ligand association is the generation of signaling cascades (this is outside-in signaling) inducing rapid spreading (Abram and Lowell, 2009). This was demonstrated not only with LFA-1 (Feng et al., 2012) but also β1 (Zeller et al., 2010) or β3 (Kasirer-Friede et al., 2007) integrins.

*In conclusion*, LFA-1-mediated leukocyte arrest on endothelial cells is a key step of inflammation. This strongly depends on a combination of integrin-mediated processes that are likely to involve a network of tens of proteins and hundreds of interactions (Ley et al., 2007; Zaidel-Bar et al., 2007). A possible way of understanding the functions of these networks is to analyze the perturbation (Ku et al., 2012) generated by the deficiency of a specific component. A recently characterized defect of *FERMT3* gene resulting in abnormal or absent kindlin-3 protein provides a good example.

## Leukocyte Adhesion Deficiency Type III Exemplifies the Consequences of a Specific Integrin Deficiency

Leukocyte adhesion deficiency (LAD) type I was identified three decades ago as a syndrome caused by a partial or complete defect of integrin β2 chain expression. Symptoms included sensitivity to bacterial infection, leukocytosis, and absence of pus formation. In 1997, a functional β2-integrin deficiency associated with a bleeding tendency and abnormal platelet spreading was reported in a patient suffering symptoms resembling but somewhat milder than LAD-I (Kuijpers et al., 1997). This was called LAD-I/variant or LAD-III (Alon and Etzioni, 2003). It was ascribed to a defective expression of kindlin-3 (Kuijpers et al., 2009). Kindlin-3 is expressed on hematopoietic cells and binds the C-terminal NXXY/F site of integrin β2 chain, thus stabilizing active conformations together with talin (Abram and Lowell, 2009). Kindlin-3 overexpression induced integrin clustering (Feng et al., 2012). Also, Kindlin-3 participates integrin-mediated cell spreading, which is considered as a consequence of outside-in signaling (Abram and Lowell, 2009; Meves et al., 2009).

Analyzing the function of kindlin-3-defective cells might give valuable information on both the role of kindlin-3 in integrin function and the interrelation of the physical events described in this review. We used the availability of a LAD-III patient to investigate neutrophils and T lymphocytes: we quantified three steps of the arrest sequence triggered by several integrin activators (Robert et al., 2011): (i) cell adhesion to ICAM-1-coated surfaces was monitored in a low shear flow (20 s^−1^). Under these conditions a single molecular bond could induce a detectable arrest (Pierres et al., 2008a), and the *total arrest frequency* should thus reflect the presence of extended integrins. (ii) The *frequency of durable arrests* (2 min or more) should account for a combination of integrin clustering and high affinity state acquisition. (iii) Finally, the *molecular contact area* between leukocytes and ICAM-1-coated surfaces after 15 min interaction, was used as a reporter of membrane-surface alignment and *spreading*. Cells were stimulated with Mn^++^, which stabilizes active conformations without involving intracellular cascades, or chemotactic peptide fMLF, phorbol myristate acetate (PMA), and calcium ionophore ionomycin that are know to activate cells by triggering signaling cascades. The following conclusions were obtained:

(i) A clear hierarchy of measured parameters was obtained: spreading could not be normal if durable arrest frequency was normal, and durable arrest could no be stimulated in patients’ cells if total arrests were lacking.
(ii) As expected, Mn^++^-induced arrests were normal in patients cells, validating the possibility of detecting individual interactions provided integrin unbending was correctly induced.
(iii) Total arrest frequency was normal, but durable arrest frequency was decreased in fMLF-stimulated neutrophils, confirming the importance of active cell functions to stabilize arrests in contrast with short-term molecular interactions (Pierres et al., 1994). This is consistent with the hypothesis that the integrin extension induced by fMLF (Diamond and Springer, 1993; El Azreq et al., 2011) might be obtained in absence of kindlin-3. It was not feasible to ascribe the arrest stabilization and spreading defects to incomplete integrin activation or defect of fMLF-induced clustering (Detmers et al., 1987). Interestingly, Lefort et al. (2012) used a murine model to compare the consequences of talin and kindlin-3 deficiencies: they concluded that talin was sufficient to trigger integrin extension and enable slow rolling, but synergy with kindlin-3 was required to induce high affinity conformation and cell arrest under flow.
(iv) Phorbol myristate acetate (PMA) was reported to induce both mobility changes (Kucik et al., 1996) and at least partial affinity increase (Lollo et al., 1993) in stimulated leukocytes. PMA treatment triggered normal arrest frequency and duration in patients’s neutrophils while T lymphocytes were markedly defective for both parameters. This is in line with a previous finding that a same pharmacological treatment had opposing effects on lymphocyte and neutrophil integrins (Marwali et al., 2003; Solomkin et al., 2007; Abram and Lowell, 2009).
(v) Surprisingly, T lymphocytes from LAD-III patients displayed abnormal spreading on anti-CD3-coated surfaces. Interestingly, LFA-1 was recently found to be involved in T lymphocyte activation by anti-CD3-coated surfaces, even in absence of ICAM-1 (Li et al., 2009) and kindlin-3 was found to lower the threshold for NK cell activation (Gruda et al., 2012). This suggests additional roles for kindlin-3. In contrast to our results, Feigelson et al. (2011) found normal ICAM-1-independent spreading of T lymphocytes from another LAD-III patient. That different kindlin-3-defective cell populations might display different deficiencies is in line with a report showing that only one of two LAD-III siblings suffered osteopetrosis (Jurk et al., 2010). Gene-gene interactions may provide a likely explanation for phenotypic differences between two subjects or cell population sharing a common genetic deficiency (Cordell, 2009).

## Conclusion

The recent expansion of molecular biology techniques allowing high throughput analysis of gene sequence and expression in individuals makes it a prominent goal to define with maximum accuracy the function of newly characterized genes and proteins. This is a formidable task due to the complexity of molecular networks driving cell functions. The current challenge is to find tractable ways of analyzing these networks. A possible way of approaching this goal may consist of determining the functional consequences of a well defined network perturbation (Ku et al., 2012). As shown in this review, recent studies provided a quantitative description of the elementary physical processes contributing cell functions (e.g., molecule clustering, molecular interactions, conformational change all contribute integrin-mediated adhesion). This should allow us to draw networks connecting these elementary physical events. These “function networks” should be simpler than the huge networks involving hundreds of molecular components and interactions that are presently available (Zaidel-Bar et al., 2007). The analysis of well defined genetic defects found in patients may be very informative in this domain.

## Conflict of Interest Statement

The authors declare that the research was conducted in the absence of any commercial or financial relationships that could be construed as a potential conflict of interest.
